# Exploring social activity patterns among community-dwelling older adults in South Korea: a latent class analysis

**DOI:** 10.1186/s12877-024-05287-5

**Published:** 2024-08-21

**Authors:** Jiyoung Shin, Hun Kang, Seongmi Choi, JiYeon Choi

**Affiliations:** 1 Mo-Im Kim Nursing Research Institute, Yonsei University College of Nursing, 50-1 Yonsei-ro, Seodaemun-gu, Seoul, 03722 South Korea; 2 Department of Social and Behavioral Sciences, Yale University School of Public Health, 60 College Street, New Haven, CT 06510 USA; 3 Yonsei University Institute for Innovation in Digital Healthcare, 50-1 Yonsei-ro, Seodaemun-gu, Seoul, 03722 South Korea; 4 Health Insurance Research Institute, National Health Insurance Service, 2, Segye-ro, Wonju-si, Gangwon-Do, 26464 South Korea

**Keywords:** Older adults, Social activity, Digital activity, Digital and in-person, Depression, Mental health, Latent class analysis, South Korea

## Abstract

**Background:**

With the trend of digitalization, social activities among the older population are becoming more diverse as they increasingly adopt technology-based alternatives. To gain a comprehensive understanding of social activities, this study aimed to identify the patterns of digital and in-person social activities among community-dwelling older adults in South Korea, examine the associated factors, and explore the difference in depressive symptoms by the identified latent social activity patterns.

**Methods:**

Data were extracted from a nationwide survey conducted with 1,016 community-dwelling older adults (mean age 68.0 ± 6.5 years, 47.8% male). The main variables assessed were digital social activities (eight items), in-person social activities (six items), and depressive symptoms (20 items). Data were analyzed using latent class analysis, multinomial logistic regression, and multiple linear regression.

**Results:**

We identified four distinct social activity patterns: “minimal in both digital and in-person” (22.0%), “moderate in both digital and in-person” (46.7%), “moderate in digital & very high in in-person” (14.5%), and “high in both digital and in-person” (16.8%). Younger age, living in multi-generational households, and higher digital literacy were associated with a higher likelihood of being in the “moderate in both digital and in-person” than the “minimal in both digital and in-person” group. Younger age, male, living in multi-generational households, residing in metropolitan areas, no dependency on IADL items, doing daily physical exercise, and higher digital literacy were associated with a higher likelihood of being in the “moderate in digital & very high in in-person” than the “minimal in both digital and in-person” group. Younger age, living in multi-generational households, no dependency on IADL items, doing daily physical exercise, and higher digital literacy were associated with a higher likelihood of being in the “high in both digital and in-person” than the “minimal in both digital and in-person” group. Depressive symptoms were significantly higher in the group with minimal engagement in both digital and in-person activities, compared to other three groups.

**Conclusions:**

This study highlights distinct patterns of social activities among Korean community-dwelling older adults. Since older adults with minimal social activity engagement can be more vulnerable to depressive symptoms, interventions that address modifiable attributes, such as supporting digital literacy and facilitating physical activity of older adults, could serve as potential strategies to enhance their social activity engagement and, consequently, their mental well-being.

## Background

With increasing digitalization, social activities among the older population are becoming diverse. More older adults increasingly adopt technology-based alternatives [1]. Notably, older adults in South Korea show high rates of smartphone usage (90%) [1] and internet usage rate (98%), surpassing those of other developed countries such as Japan, Sweden, and the Netherlands [1, 2]. Current social activities now encompass digital interactions including social media use [3], complementing in-person social interactions [4]. Therefore, to gain a deeper understanding of social activities among older individuals in our rapidly digitizing society, it is crucial to explore both traditional, predominantly in-person social activities and digital-based remote interactions.

Studies have consistently demonstrated that engaging in social activities can help prevent and alleviate depressive symptoms in older adults [5–11]. In a large cohort study in the United States, perceived social activity level was identified as the social determinant most strongly associated with depression among older adults [12]. Additionally, social participation, including social connections, informal social participation, and volunteering, has been linked to promoting healthy aging and reducing depressive symptoms in older adults [13]. However, the direction and strength of the association between social activity and depression may vary depending on the types of social activity [12].

While previous studies have mainly focused on exploring the effect of each social activity on depression individually [14–17], only a few studies classified social activities and compared the characteristics based on patterns of social activities [18, 19]. Since we tend to engage in social activities in a complex manner rather than exclusively choosing one, we need to consider the possibility that their individual effects may offset each other [15]. Considering all, it is valuable to examine the factors associated with social activities to gain specific and personalized insights into modifiable interventions.

Furthermore, despite the increasing digitalization, most previous studies investigating the relationship between social activities and depressive symptoms have primarily focused on either digital or in-person modes of social activities [5–8, 10]. To gain a comprehensive understanding of how older adults participate in various social activities, including both digital and in-person activities, it is essential to explore the combined engagement in these diverse forms of social activities. This is particularly important within the context of an aging population in a rapidly digitalizing world. Therefore, this study aimed to (1) identify the patterns of digital and in-person social activities among community-dwelling older adults in South Korea, (2) identify the associated factors, and (3) explore the difference in depressive symptoms by the identified latent social activity patterns.

## Methods

### Data source and sample

Data were extracted from a nationwide cross-sectional survey conducted to understand the digital literacy status and associated factors among community-dwelling older adults in South Korea [20]. The survey was conducted from October to November 2022, using proportional stratified sampling based on region, sex, and age groups to match South Korea’s registered population in June 2022. Participants who met the following criteria were selected: (1) aged 60 or older, (2) achieved a minimum score of 22 on the Korean version of the Mini-Mental State Examination (2nd edition) [21], and (3) proficient in the Korean language. A total of 1,016 older adults participated and provided information on their sociodemographic characteristics, health status, health behavior, social activities, and social support.

### Ethics approval and consent to participate

This study was reviewed and approved by the Institutional Review Board of Yonsei University (ref no.: 4-2023-0983). Participants provided informed consent before the survey and received gift vouchers worth 10,000 Korean won as compensation upon completing survey.

### Measures

#### Social activities

Social activities encompassed both traditional in-person social activities and digital social activities. In-person social activities were evaluated via questions adapted from the Korea Longitudinal Study of Ageing (KLoSA) [22]. Participants were asked regarding the frequency of their involvement in various activities over the past year, such as religious gatherings, social gatherings, leisure/culture/sports activities, alumni meetings, volunteer work, political/civic/interest group activities, and others. Responses that indicated “not at all” were recoded as non-participation (0), while other responses were categorized as participation (1) for six social activities, which excluded the “others” category.

To assess digital social activities—which included phone calls, text messages, messengers, information search, e-mails, blogs, online education, and app use—, participants were asked regarding the purpose of their digital device use which included desktops and laptops, mobile phones, tablets, e-books, and wearable devices. Options included phone calls, text messages, messengers, information search, e-mail, blogs, online education, other, or not using digital devices. Variables for digital social activities were coded as participation (1) or non-participation (0) for each activity, which excluded the “other” and “not using digital devices.”

#### Depressive symptoms

The frequency and severity of depressive symptoms were measured via the Center for Epidemiological Studies-Depression (CES-D) scale which consists of 20 items [23]. We used the integrated Korean version of the CES-D developed by Jeon et al. [24]. Participants rated their experience of depressive symptoms over the past week on a 0–3 scale for each item, which ranged from “rarely or never (< 1 day)” to “most or all of the time (5–7 days).” After four positively worded items were reverse-coded, we aggregated the scores. The total scores ranged between 0 and 60, and higher scores indicated more severe depressive symptoms [23]. We also categorized respondents as either at risk for clinical depression or not, using the most widely recommended threshold (≥ 16) [25]. The original CES-D and Korean version had Cronbach’s alphas of 0.85 and 0.91, respectively [23, 24]. In our sample, the Cronbach’s alpha was 0.89.

#### Variables associated with social activity

##### Socio-demographic factors

Based on previous studies [19, 26–29], participants’ sociodemographic factors included age, sex, educational level, living arrangements, region, and economic activities. Age was recorded in years, and sex was coded as male (1) or female (0). Educational levels were categorized as below middle school (0), below high school (1), below college (2), and college or higher (3). Living arrangements were categorized as living alone (0), with a spouse (1), or in a multigenerational household (2). Region was divided into metropolitan areas (1), which included Seoul, Incheon, and Gyeonggi provinces, and non-metropolitan areas (0), which included cities and provinces outside the metropolitan area. Economic activities were coded as “Yes” (2) when currently engaged in income-generating work, “Used to” (1) when previously employed but not currently, and “Never” (0) when there has been no lifetime work experience.

##### Health factors

Variables that may be associated with social activity were selected based on previous studies [27–30]. Health status variables included diagnosed disabilities, chronic diseases, functional status, and health-related quality of life. Participants indicated whether they had been diagnosed with a disability (1) or not (0). To assess older adults’ objective health status, participants reported whether they had been diagnosed with a chronic condition that lasted over three months [31]. The number of chronic diseases was categorized as 0, 1, and 2 or more.

Functional status was assessed using the Korean Activities of Daily Living (K-ADL) and Korean Instrumental Activities of Daily Living (K-IADL). The K-ADL and K-IADL consist of seven items (dressing, washing face and hands, bathing, eating, transfer, toileting, and continence) and 10 items (decorating, housework, preparing meals, laundry, going out for a short distance, using transportation, shopping, handling money, using the telephone, and taking medicine), respectively [32]. Participants answered each item based on the extent to which they required assistance. The Cronbach’s alpha values for the K-ADL and K-IADL at the time of development were 0.94 [33] and 0.94 [34], respectively. In our sample, the Cronbach’s alpha values were 0.75 and 0.78, respectively. For analysis, we categorized the number of items that required assistance into three groups: 0, 1, and 2 or more.

Health-related quality of life was assessed via the 12-item Short-Form Health Survey (SF-12), a concise version of the 36-item Short-Form Health Survey [35], version 2 (SF-12v2). The SF-12 measures eight health domains: physical functioning, role-physical, bodily pain, general health, vitality, social functioning, role-emotional, and mental health. The scores for each domain contributed to the Physical Component Summary (PCS) and Mental Component Summary (MCS) scores. Scores for the PCS (six items) and MCS (six items) were calculated and standardized based on published algorithms for the SF-12v2 [35]. On a range of 0–100, higher scores indicated a higher quality of life. In the original version of the SF-12, the Cronbach’s alpha values for PCS and MCS were 0.89 and 0.86 for the United States and 0.76 and 0.77 for the United Kingdom, respectively [35]. In our sample, the Cronbach’s alpha values were 0.81 for PCS and 0.72 for MCS.

Health behaviors included smoking, alcohol consumption, and physical activity. Based on their smoking status, participants were categorized as non-smoker (0), ex-smoker (1), or current smoker (2). Regarding alcohol consumption, participants answered by specifying whether they had consumed alcohol at least once during the past year (1) or not (0). Physical activity was assessed based on whether participants engaged in continuous physical activity for 10 min or more (1) or not (0).

##### Social factors

Digital literacy and social support were chosen as potential social factors associated with social activity, based on prior findings [5]. Digital literacy was assessed via the Everyday Digital Literacy Questionnaire (EDLQ) developed from the survey of our data source. The EDLQ was created with reference to the European Commission’s Digital Competence (DigComp) framework and consists of three domains: information and communication (nine items), content creation and management (four items), and safety and security (nine items) [20]. Participants responded to each item on a 5-point Likert scale that ranged from 1 (not at all) to 5 (very much so). Higher scores indicated higher levels of digital literacy. The EDLQ exhibited a high level of reliability with a Cronbach’s alpha value of 0.98 [20].

Social support was assessed via the Multidimensional Scale for Perceived Social Support (MSPSS), which evaluated perceived social support from family, friends, and significant others on 12 items [36]. The original instrument was structured with a 7-point Likert scale, which ranged from 1 (very strongly disagree) to 7 (very strongly agree) [36]. For this present study, we opted to use the Korean translated version, which employed a 5-point Likert scale that ranged from “strongly disagree” to “strongly agree” [37], in consideration of both the translated version we referenced and our participants’ characteristics. A higher mean score indicated a greater level of perceived social support. The Cronbach’s alpha values were 0.88, 0.89, and 0.94 for the original version, Korean translated version, and our sample, respectively.

### Data analysis

Latent Class Analysis (LCA) is a person-centered modeling approach that relies on the response patterns of observed variables to identify latent subpopulations within a sample [38]. This approach can be particularly valuable to identify multiple subgroups within a sample that share common characteristics and could benefit from similar interventions [38]. We employed a LCA to identify patterns of social activity among community-dwelling older adults, using six in-person activities and eight digital social activities as indicator variables for social activities. The sample size was considered adequate, aligning with the recommendations of numerous prior studies that suggested the inclusion of 300 or more participants [39].

Model fit was assessed via three information criteria: Bayesian Information Criterion (BIC), Akaike Information Criterion (AIC), and Sample-Size Adjusted Bayesian Information Criterion (SSABIC). Lower values indicated a better fit [38]. To determine how accurately the model defined the classes, we employed entropy [40]. An entropy value of 0.8 or higher was recommended as an acceptable threshold, with values closer to 1 considered ideal [41]. Class solutions were evaluated via three relative fit indices: Lo-Mendell-Rubin Likelihood Ratio Test (LMR-LRT), Adjusted Lo-Mendell-Rubin Likelihood Ratio Test (Adj. LMR-LRT), and Bootstrapped Likelihood Ratio Test (BLRT). These indices assessed whether a model with k classes significantly improved the fit compared with a model with k-1 classes [42]. If the improvement was not statistically significant (*p* > .05), the model with k-1 classes was selected [42].

Next, variables associated with social activities were introduced as auxiliary variables to minimize classification errors among classes, which resulted in the creation of the most probable class variables [43]. After then, we identified variables that differentiated the classes via a multinomial logistic regression model. We estimated the odds ratio (OR) for the likelihood of belonging to a specific class membership compared with the reference group, along with their corresponding 95% confidence intervals (CI). Additionally, we conducted an analysis of variance (ANOVA) to compare depressive symptoms among classes derived from LCA. Finally, we examined the association between depressive symptoms and social activity classes using multiple linear regression. In our analysis, we controlled for covariates known to influence depressive symptoms in older adults, as identified in previous reviews [44–48]. The LCA and multinomial logistic regression were conducted using Mplus 8.8 (Muthén & Muthén) [43] and subsequent analyses were performed using IBM SPSS Statistics for Windows version 26.0 (IBM Corp., Armonk, NY, USA).

## Results

### Sample characteristics

Table 1 shows participants’ characteristics. The mean age of 1,016 participants was 68.0 years (SD = 6.5). More than half of the participants were female (52.2%, *n* = 530), had completed education beyond high school graduation (54.2%, *n* = 551), lived with spouse (51.6%, *n* = 524), and resided in non-metropolitan areas (53.8%, *n* = 547). Regarding physical health, 95.2% (*n* = 967) reported having no disability, and 69.3% (*n* = 704) reported having diagnosis of one or more chronic condition.

**Table 1:** Sample characteristics (*n* = 1,016)

Variables	M(SD) or *n*(%)
Age (years)	68.0 (6.5)
Sex	
Male	486 (47.8)
Female	530 (52.2)
Education level	
Below middle school	240 (23.6)
Below high school	225 (22.1)
Below college	451 (44.4)
College and above	100 (9.8)
Living arrangements	
Living alone	199 (19.6)
Living with a spouse	524 (51.6)
Multi-generational households	293 (28.8)
Region	
Metropolitan area ^a^	469 (46.2)
Non-metropolitan area ^b^	547 (53.8)
Engaged in economic activities	
Never	40 (3.9)
Used to	354 (34.8)
Yes	622 (61.2)
No. of diagnosed chronic diseases	
0	312 (30.7)
1	301 (29.6)
≥ 2	403 (39.7)
Disability	
No	967 (95.2)
Yes	49 (4.8)
No. of dependent items of K-ADL	
0	952 (93.7)
1	37 (3.6)
≥ 2	27 (2.7)
No. of dependent items of K-IADL	
0	839 (82.6)
1	68 (6.7)
≥ 2	109 (10.7)
HRQoL (scores)	
PCS	50.1 (7.3)
MCS	49.4 (8.4)
Exercise	
No	422 (41.5)
Yes	594 (58.5)
Alcohol consumption	
No	516 (50.8)
Yes	500 (49.2)
Smoking	
Non-smoker	628 (61.8)
Ex-smoker	260 (25.6)
Current-smoker	128 (12.6)
EDLQ-22 (scores)	57.1 (24.0)
MSPSS (scores)	3.9 (0.7)
CES-D (scores)	12.0 (7.6)
1–15	750 (73.8)
≥ 16	266 (26.2)

Note. M: Mean; SD: Standard deviation; K-ADL: Korean Activities of Daily Living; K-IADL: Korean-Instrumental Activities of Daily Living; HRQoL: Health-Related Quality of Life; PCS: Physical Component Summary; MCS: Mental Component Summary; EDLQ-22: Everyday Digital Literacy Questionnaire 22-item; MSPSS: Multidimensional Scale of Perceived Social Support; CES-D: Center for Epidemiological Studies-Depression.

^a^ Metropolitan area: Seoul, Incheon, and Gyeonggi province

^b^ Non-metropolitan area: cities and provinces other than the metropolitan area

The results of K-ADL and K-IADL indicate that 93.7% (*n* = 952) had no dependence in ADL and 82.6% (*n* = 839) had no dependence in any of IADL. More than half of the participants reported being engaged in daily physical exercise for more than 10 min per day (58.5%, *n* = 594), were non-drinkers (50.8%, *n* = 516) and non-smokers (61.8%, *n* = 628). Health related quality of life scores for physical and mental health were 50.1 ± 7.3 and 49.4 ± 8.4 respectively. Digital literacy had a mean score of 57.1 ± 24.0, and perceived social support had a mean score of 3.9 ± 0.7. The mean score for depressive symptoms across our sample was 12.0 ± 7.6 and 26.2% (*n* = 266) were at risk for clinical depression.

### Identification of social activity patterns

#### Model selection: 4-class model

Table 2 summarizes the model fit indices for the selectable 2 to 6 latent classes. Decreases in the AIC, BIC, and SSABIC values were less pronounced after the 3-class point. The entropy values, which indicated the quality of the class classification, satisfied the recommended thresholds across all the models. The *p*-values for the LMR-LRT and Adj. LMR-LRT were not statistically significant (*p* < .05) in the 5-class and 6-class scenarios. Considering the strong statistical evidence of the goodness-of-fit measures and the theoretical interpretability, the 4-class model was chosen for our sample.

**Table 2:** Summary of the model fit indices for the latent class analysis

Fit Indices	1-class	2-class	3-class	4-class ^a^	5-class	6-class
AIC	13938.376	12049.35	11477.40	**11281.67**	11122.21	11046.63
BIC	14007.307	12192.14	11694.04	**11572.17**	11486.56	11484.84
SSABIC	13962.841	12100.03	11554.29	**11384.78**	11251.53	11202.17
ENTROPY		0.878	0.813	**0.822**	0.807	0.829
LMR-LRT *p*-value		< 0.0001	< 0.0001	**0.0234**	0.0673	0.1374
Adj. LMR-LRT *p*-value		< 0.0001	< 0.0001	**0.0241**	0.0686	0.1393
BLRT *p*-value		< 0.0001	< 0.0001	**< 0.0001**	< 0.0001	< 0.0001

Note. AIC: Akaike Information Criterion; BIC: Bayesian Information Criterion; SSABIC: Sample-Size Adjusted Bayesian Information Criterion; LMR-LRT: Lo-Mendell-Rubin Likelihood Ratio Test; Adj. LMR-LRT: Adjusted Lo-Mendell-Rubin Likelihood Ratio Test; BLRT: Bootstrapped Likelihood Ratio Test

^a^ Selected class solution is in bold

#### Characterization of social activity patterns

The characteristics of social activity patterns in the 4-class model can be explored based on the probabilities (ranging from 0 to 1) of respondents indicating participation in each social activity (see Table 3). Final class frequencies and proportions for the latent classes were described based on their most likely latent class membership. Class 1, the second-largest group (22.0%, *n* = 224), showed the lowest level of participation in overall social activities among the four classes. Approximately half participated in phone calls (49.2%) and about a quarter used text messages (24.0%), while the likelihood of participation in other digital device activities was very limited. For in-person social activities, over half had a probability of participating in social gatherings (55.1%); however, the probability of participation in other activities remained at the lowest level among the four classes.

**Table 3:** Probability scale in each social activity by class

Variables		Class 1: Minimal in both digital and in-person (*n* = 224)	Class 2: Moderate in both digital and in-person (*n* = 474)	Class 3: Moderate in digital & very high in in-person (*n* = 147)	Class 4: High in both digital and in-person (*n* = 171)
		P (SE)	P (SE)	P (SE)	P (SE)
Digital social activity	Phone calls	0.492 (0.038)	0.995 (0.005)	0.965 (0.019)	1.000 (0.000)
	Text messages	0.240 (0.034)	0.982 (0.010)	0.882 (0.038)	0.988 (0.009)
	Messengers	0.028 (0.016)	0.958 (0.014)	0.933 (0.035)	0.989 (0.008)
	Information search	0.029 (0.014)	0.802 (0.027)	0.832 (0.056)	1.000 (0.000)
	Email	0.000 (0.000)	0.090 (0.024)	0.146 (0.094)	0.745 (0.073)
	Blogs	0.000 (0.000)	0.060 (0.017)	0.076 (0.078)	0.531 (0.074)
	Online Education	0.014 (0.009)	0.187 (0.031)	0.254 (0.050)	0.797 (0.071)
	App use	0.000 (0.000)	0.056 (0.023)	0.066 (0.031)	0.716 (0.086)
In-person social activity	Religious gatherings	0.160 (0.026)	0.186 (0.021)	0.360 (0.063)	0.315 (0.041)
	Social gatherings	0.551 (0.035)	0.751 (0.028)	1.000 (0.000)	0.901 (0.030)
	Leisure/culture/sports activities	0.160 (0.028)	0.092 (0.031)	0.801 (0.087)	0.481 (0.071)
	Alumni meetings	0.187 (0.029)	0.288 (0.040)	0.917 (0.055)	0.719 (0.065)
	Volunteer work	0.026 (0.011)	0.028 (0.010)	0.381 (0.111)	0.194 (0.063)
	Political/civic/interest group activities	0.000 (0.000)	0.002 (0.002)	0.224 (0.098)	0.109 (0.054)

Note. P, Probability; SE, Standard error

Class 2, the largest group (46.7%, *n* = 474), displayed notable variation in participation rates across different types of social activities. Regarding digital social activities, majority had participated in phone calls (99.5%), text messages (98.2%), messenger apps (95.8%), and information search (80.2%). However, participation in other activities remained low (< 20%). Regarding in-person social activities, apart from social gatherings (75.1%) and alumni meetings (28.8%), participation rates were consistently below 20%.

Class 3, the smallest group (14.5%, *n* = 147), exhibited similar likelihoods of participating in digital social activities compared to Class 2, while displaying the highest likelihood of participating in in-person social activities among the four groups. Respondents in Class 3 showed a high likelihood of participating not only in social gatherings (100.0%) but also in alumni meetings (91.7%) and leisure/culture/sports activities (80.1%).

Class 4, the third largest group (16.8%, *n* = 171), showed the highest likelihood of engagement in digital social activities among the four groups, and also demonstrated the second highest level of participation in in-person social activities. Particularly, the majority of respondents in Class 4 indicated a likelihood of participation in phone calls (100.0%), information search (100.0%), messenger apps (98.9%), and text messages (98.8%), with over 50% indicating participation in the remaining digital activities as well.

**Fig. 1:**
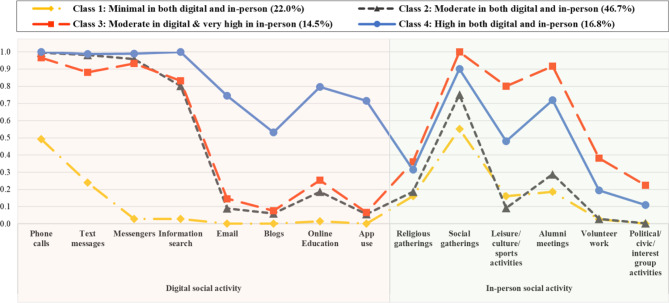
Probability of the social activities among the four latent classes

Figure 1 illustrates the probability of social activity for each class. The x-axis and y-axis display indicators of social activities and a probability scale from 0 to 1, indicating the probability of engaging in each activity. Based on the distribution of the 14 social activities, classes were labeled as “minimal in both digital and in-person” (Class 1, 22.0%, *n* = 224), “moderate in both digital and in-person” (Class 2, 46.7%, *n* = 474), “moderate in digital & very high in in-person” (Class 3, 14.5%, *n* = 147), and “high in both digital and in-person” (Class 4, 16.8%, *n* = 171).

### Characteristics associated with social activity patterns

Table 4 presents the results of multinomial logistic regression examining the role of characteristics that may be associated with social activity patterns. Younger age, multi-generational households compared to living alone, and higher digital literacy were associated with a higher likelihood of being in the “moderate in both digital and in-person” social activity group rather than the “minimal in both digital and in-person” group. In addition, younger age, male, multi-generational households, residing in metropolitan areas, no dependency on IADL items, doing daily physical exercise for more than 10 min, and higher digital literacy were associated with a higher likelihood of being in the “moderate in digital & very high in in-person” social activity group rather than the “minimal in both digital and in-person” group. Similarly, younger age, multi-generational households, no dependency on IADL items, doing daily physical exercise for more than 10 min, and higher digital literacy were associated with a higher likelihood of being in the “high in both digital and in-person” social activity group rather than the “minimal in both digital and in-person” group.

**Table 4:** Multinomial logistic regression for associated variables of social activity patterns

Variables	“Moderate in both digital and in-person” vs “Minimal in both digital and in-person” (ref)		“Moderate in digital & very high in in-person” vs “Minimal in both digital and in-person” (ref)		“High in both digital and in-person” vs “Minimal in both digital and in-person” (ref)	
	OR	95% CI	OR	95% CI	OR	95% CI
Age (years)	0.92***	0.87–0.96	0.90**	0.84–0.96	0.85**	0.77–0.94
Sex (ref: Female)						
Male	1.46	0.66–3.24	3.24*	1.13–9.29	2.12	0.52–8.67
Education level (ref: College or higher)						
Below middle school	1.61	0.39–6.63	0.37	0.07–2.07	0.21	0.03–1.60
Below high school	3.35	0.83–13.56	1.20	0.26–5.55	0.15	0.02–1.32
Below college	3.41	0.75–15.39	1.64	0.34–7.91	0.57	0.10–3.20
Living arrangements (ref: Multi-generational households)						
Living alone	0.33**	0.15–0.72	0.22*	0.07–0.77	0.23*	0.06–0.82
Living with a spouse	0.58	0.28–1.20	0.64	0.26–1.59	0.58	0.20–1.67
Region (ref: Non-metropolitan area)						
Metropolitan area	1.03	0.57–1.87	2.09*	1.03–4.26	2.22	0.91–5.42
Engaged in economic activities (ref: Yes)						
Never	0.70	0.22–2.22	1.80	0.40–8.17	2.48	0.45–13.75
Used to	0.59	0.32–1.07	0.99	0.44–2.20	1.25	0.47–3.36
No. of diagnosed chronic diseases (ref: ≥ 2)						
0	0.70	0.33–1.50	0.62	0.25–1.59	0.90	0.29–2.80
1	1.13	0.53–2.41	0.71	0.28–1.81	1.10	0.36–3.36
Disability (ref: No)						
Yes	1.42	0.46–4.34	1.14	0.18–7.37	1.90	0.18–19.64
No. of dependent items of K-ADL (ref: ≥ 2)						
0	5.32	0.38–74.87	1.99	0.10-40.66	2.34	0.09–60.98
1	3.16	0.03-323.94	1.16	0.01-157.64	0.86	0.01-134.67
No. of dependent items of K-IADL (ref: ≥ 2)						
0	1.78	0.61–5.18	7.27**	1.72–30.83	22.28*	1.69-294.63
1	0.71	0.13-4.00	2.34	0.24–22.84	5.90	0.25-141.24
HRQoL (scores)						
PCS	0.98	0.94–1.02	0.97	0.91–1.02	1.00	0.92–1.09
MCS	0.99	0.95–1.03	0.97	0.93–1.02	0.96	0.90–1.02
Exercise (ref: No)						
Yes	1.55	0.86–2.81	3.23**	1.46–7.14	3.05*	1.21–7.68
Alcohol consumption (ref: No)						
Yes	1.09	0.58–2.05	1.22	0.55–2.70	0.98	0.38–2.52
Smoking (ref: Current-smoker)						
Non-smoker	2.28	0.84–6.21	2.54	0.76–8.54	1.79	0.39–8.30
Ex-smoker	1.45	0.48–4.40	1.30	0.35–4.81	1.85	0.43–8.02
EDLQ-22 (scores)	1.12***	1.09–1.15	1.13***	1.10–1.17	1.23***	1.18–1.29
MSPSS (scores)	1.05	0.69–1.60	1.34	0.76–2.36	1.78	0.88–3.61

Note. OR: Odds ratio; CI: Confidence interval; K-ADL: Korean Activities of Daily Living; K-IADL: Korean-Instrumental Activities of Daily Living; HRQoL: Health-Related Quality of Life; PCS: Physical Component Summary; MCS: Mental Component Summary; EDLQ-22: Everyday Digital Literacy Questionnaire 22-item; MSPSS: Multidimensional Scale of Perceived Social Support

* *p* < .05, ** *p* < .01, *** *p* < .001

### Comparison of depressive symptoms by social activity patterns

Table 5 shows the results of the ANOVA conducted to explore the differences in depressive symptoms by identified social activity patterns. The mean CES-D scores were observed as follows: minimal in both digital and in-person (14.64 ± 8.52), moderate in both digital and in-person (11.67 ± 7.24), moderate in digital & very high in in-person (11.21 ± 7.41), and high in both digital and in-person (10.03 ± 6.83), with significant differences among the groups.

**Table 5:** ANOVA of depressive symptoms by social activity patterns

Social activity patterns	CES-D (scores)			
	M	SD	F	*p*
Minimal in both digital and in-person	14.64	8.52	14.03	< .001
Moderate in both digital and in-person	11.67	7.24		
Moderate in digital & very high in in-person	11.21	7.41		
High in both digital and in-person	10.03	6.83		

Note. M: Mean; SD: Standard deviation; CES-D: Center for Epidemiological Studies-Depression

Subsequently, we examined the association between depressive symptoms and social activity patterns, controlling for sex, chronic diseases, disabilities, functional status, physical activity, and alcohol consumption. There was no multicollinearity among the variables included in the model (variance inflation factor < 10). After accounting for covariates, the association between social activity patterns and depressive symptoms remained significant (see Table 6). Compared to the group with minimal engagement in both digital and in-person activities, all three other groups exhibited disparities in the CES-D scores.

**Table 6:** Association between depressive symptoms and social activity patterns

	β	SE	*p*	VIF
Social activity patterns (ref: Minimal in both digital and in-person)				
Moderate in both digital and in-person	-1.58	0.81	.051	1.57
Moderate digital & very high in in-person	-2.06	0.80	.010	1.75
High in both digital and in-person	-1.49	0.62	.016	1.84
Sex (ref: Female)				
Male	-0.72	0.51	.157	1.25
No. of diagnosed chronic diseases (ref: ≥ 2)				
0	-2.76	0.57	< .001	1.33
1	-1.98	0.56	< .001	1.29
Disability (ref: No)				
Yes	1.21	1.12	.281	1.13
No. of dependent items of K-ADL (ref: ≥ 2)				
0	-4.84	1.60	.003	2.94
1	-3.02	1.94	.120	2.57
No. of dependent items of K-IADL (ref: ≥ 2)				
0	-2.92	0.85	< .001	2.03
1	-2.17	1.17	.063	1.66
Exercise (ref: No)				
Yes	-1.40	0.47	.003	1.05
Alcohol consumption (ref: No)				
Yes	-0.29	0.50	.564	1.21
F (*p*)	10.348 (< .001)			
R^2^	0.118			
Adjusted R^2^	0.107			

Note. SE: Standard error; VIF: Variance Inflation Factor; K-ADL: Korean Activities of Daily Living; K-IADL: Korean-Instrumental Activities of Daily Living

## Discussion

### Principal findings

We investigated social activity patterns among a nationwide sample of community-dwelling older adults in South Korea. Highlighting the heterogeneity and diversity of social activity within the older adult population, we examined the patterns of social activities encompassing both digital and in-person interactions. Four distinct groups emerged: “minimal in both digital and in-person,” “moderate in both digital and in-person,” “moderate in digital & very high in in-person,” and “high in both digital and in-person” social activity groups. Older adults in the minimal social activity group showed significantly higher levels of depressive symptoms compared to the other three groups, while accounting for covariates. This finding supports previous studies indicating an inverse association between older adults’ social activity and depressive symptoms [5–12]. This study builds upon prior research by identifying a subgroup of older adults characterized by significant inactivity, near isolation, and heightened levels of depressive symptoms compared to other groups within the population. The lessons learned from this study, which focuses on older adults in South Korea – where aging and digitalization are progressing at a pace unmatched elsewhere – can serve as valuable references for future digital policies and support programs for older adults in other regions worldwide experiencing similar demographic and technological shifts.

The four latent classes showed more pronounced distinctions in digital activities compared to in-person activities. While in-person social activities consistently demonstrated a high probability of participation across classes, in the order of social gatherings, alumni meetings, leisure/culture/sports activities, or religious gatherings, digital social activities displayed distinct characteristics and variations across the classes.

Interestingly, digital social activity of the two “moderate in digital” groups, comprising 61.2% of the sample, predominantly consisted of individual communication methods, such as phone calls, text messages, and messengers, as well as online information search. However, their participation rates in activities such as email, blogs, online education, and other app use were very low, like the minimally active group. This characterization is consistent with findings from a nationally representative sample from South Korea in 2020, indicating that over 89% of the sample engaged in digital social activities related to individual communication (e.g., receiving/sending messages), while involvement in more advanced activities (e.g., online commerce, app use, financial activities) remained low, at less than 20% [49]. On the other hand, the minimal group, which exhibited the highest depressive symptoms, showed notably low levels of engagement even in individual communication via mobile phones. In the minimal group, it was discovered that one out of two individuals may not participate in phone calls, and only one out of five may engage in text messaging activities. These findings suggest that despite the high smartphone use rate among Korean older adults [1] and the country’s leading global internet usage rate [2], approximately 20% of older adults may remain digitally isolated. To address this issue and assist the minimally active group reaching at least a moderately active level, interventions should be tailored to cater the diverse needs of older adults. Factors such as health status, socioeconomic status, and resource availability, which are linked to social activity, should be considered.

Our results highlight two modifiable attributes – digital literacy and physical activity – that could facilitate social activities among older adults. Specifically, in our sample, older adults with higher digital literacy were more inclined to belong to the moderately or highly active group rather than the minimal social activity group. This finding aligns with a previous study that reported a positive association between older adults’ engagement in social activities and their use of information and communications technology (ICT), particularly in relation to digital literacy [50]. In our study, it is noteworthy that older adults with high digital literacy were more likely to belong to the other moderately or highly social active group than the minimally active group, regardless of their age or living arrangements. This finding underscores the importance of developing strategies to support digital literacy and encourage the use of digital device to facilitate social activities among older adults. For example, providing digital literacy education tailored for older adults [51, 52] may enhance their ability to use digital devices for social activities. This can create opportunities for older adults to engage in social interactions, even in the absence of in-person interactions [53, 54]. Importantly, interventions targeting older adults should be tailored to their unique needs. Digital literacy training programs can be structured in tiers or personalized to individual capacities [52]. When working with older adults, it is crucial to recognize that they may require more time, patience, and frequent reminders to grasp digital skills effectively [55]. Additionally, efforts should focus on fostering positive perceptions and experiences of ICT among older adults. By accumulating positive experiences with ICT, older adults can develop a deeper understanding and curiosity about technology, seamlessly integrate it into their daily routines, and enhance their overall digital literacy [56, 57]. Such interventions can be especially valuable in unforeseen circumstances where in-person social interactions are not possible, such as during the COVID-19 pandemic. It can aid older adults in staying connected and reducing feeling of isolation [58]. Moreover, since older adults may face limitations in participating in in-person social activities due reduced physical capabilities [59], providing them with the skills to engage in digital social interactions could prove beneficial for maintaining their social and psychological well-being.

Based on our findings, physical activity also emerges as a contributing factor in fostering social activity among older adults [60, 61]. Older adults who engaged in more than 10 min of daily physical activity were more inclined to belong to the “moderate in digital & very high in in-person” group and “high in both digital and in-person” group compared to the “minimal in both digital and in-person” group. Previous studies have similarly highlighted the association between physical activity and social activity [29, 30]. This association may be partially attributed to the social aspects inherent in physical activity, where participants interact with others; hence, physical activity itself serves as a form of social activity [62, 63]. Especially, group-based physical activity classes, such as aerobic exercise, walking, and strength training [64], inherently promote social interactions among group members. Such programs are likely to sustain older adults’ involvement in social activities and, additionally, promote their psychosocial well-being and mental health. In situations where in-person gatherings are not feasible, such as during a pandemic, exchanging and discussing physical activity experiences via social networking services can also foster a sense of social connectedness [65].

Furthermore, our research indicates that older adults who are older or living alone are more inclined to be categorized into a minimally socially active group rather than the other three groups, corroborating the findings of previous studies. While further research is warranted to investigate the socioeconomic and health-related characteristics influencing older adults’ social activity participation more comprehensively, individuals with these characteristics should be given priority attention, especially considering limited community resources.

### Limitations

This study has several limitations. First, we analyzed cross-sectional data; thus, we could not consider time variables, which made it challenging to establish causal relationships among the relevant variables. Additional longitudinal studies are needed to better understand how social activities among older adults evolve over time, whether changes in depressive symptoms are associated with different patterns of social activity, and which factors influence these changes. Second, although we included six indicators of in-person social activities and eight indicators of digital social activities, it is possible that other meaningful activities were missed. Nevertheless, to our knowledge, this study is significant as one of the first comprehensive investigations on expected social activities among older adults in a digital society. Third, due to the characteristics of our analytical methodology, we dichotomized each social activity into participation and non-participation, which limited our understanding of the extent of participation in each social activity. Future research should adopt a more detailed approach by clustering social activities among older adults based on their frequency, intensity, and quality. Fourth, although we emphasize the importance of preventing depressive symptoms by promoting social activities among older adults, our study did not directly examine factors associated with depressive symptoms, as our primary focus was on social activity patterns. In our study, the social activity patterns identified through latent class analysis are unique to our sample and are not established concepts. Future studies could concentrate on analyzing the impact of more well-defined social activity patterns on depressive symptoms. This approach has the potential to provide in-depth insights into effective strategies for preventing and addressing depressive symptoms among older adults.

## Conclusions

Our findings suggest that distinct patterns of social activity can be observed among community-dwelling older adults. Furthermore, these patterns may have varying implications for the risk of depressive symptoms. Notably, older adults with limited social activity were more susceptible to depressive symptoms. Therefore, interventions that address modifiable factors, such as supporting digital literacy of older adults, could serve as a potential strategy to enhance their social engagement and, consequently, their mental well-being. Furthermore, promoting physical activity may be a promising interventional approach to encourage older adults to actively participate in social activities.

## Data Availability

The datasets used and/or analyzed during the current study are available from the corresponding author on reasonable request.

## Abbreviations

Adj. LMR-LRT: Adjusted Lo-Mendell-Rubin Likelihood Ratio Test
ADL: Activities of daily living
AIC: Akaike Information Criterion
ANOVA: Analysis of variance
BIC: Bayesian Information Criterion
BLRT: Bootstrapped Likelihood Ratio Test
CES-D: Center for Epidemiological Studies-Depression scale
CI: Confidence interval
DigComp: Digital Competence
EDLQ: Everyday Digital Literacy Questionnaire
IADL: Instrumental activities of daily living
ICT: Information and communication technology
KLoSA: Korea Longituinal Study of Ageing
LCA: Latent class analysis
LMR-LRT: Lo-Mendell-Rubin Likelihood Ratio Test
MCS: Mental Component Summary
MSPSS: Multidimensional Scale for Perceived Social Support
OR: Odds ratio
PCS: Physical Component Summary
SF-12: 12-item Short-Form Health Survey
SF-12v2: 12-item Short-Form Health Survey—version 2
SSABIC: Sample-Size Adjusted Bayesian Information Criterion

## References

[1] Gallup, Report. The 2012–2022 report on smartphone use & brand, smart watch, and wireless earphone. Gallup. 2022. https://www.gallup.co.kr/gallupdb/reportContent.asp?seqNo=1309 Accessed 12 Apr 2024.

[2] Wike R, Silver L, Fetterolf J, Huang C, Austin S, Clancy L, Gubbala S. Internet, smartphone and social media use. Pew Research Center. 2022. https://www.pewresearch.org/global/2022/12/06/internet-smartphone-and-social-media-use-in-advanced-economies-2022/ Accessed 12 Apr 2024.

[3] Hunsaker A, Hargittai E. A review of internet use among older adults. New Media Soc. 2018;20(10):3937–54. 10.1177/1461444818787348

[4] Cornejo R, Tentori M, Favela J. Enriching in-person encounters through social media: a study on family connectedness for the elderly. Int J Hum Comput Stud. 2013;71(9):889–99. 10.1016/j.ijhcs.2013.04.001

[5] Choi E, Han KM, Chang J, Lee YJ, Choi KW, Han C, Ham BJ. Social participation and depressive symptoms in community-dwelling older adults: emotional social support as a mediator. J Psychiatr Res. 2021;137:589–96. 33168196 10.1016/j.jpsychires.2020.10.043

[6] Hao G, Bishwajit G, Tang S, Nie C, Ji L, Huang R. Social participation and perceived depression among elderly population in South Africa. Clin Interv Aging. 2017;12:971–6. 28694690 10.2147/CIA.S137993 PMC5491569

[7] Jeon GS, Choi KW, Jang KS. Social networking site usage and its impact on depressive symptoms among older men and women in South Korea. Int J Environ Res Public Health. 2020;17(8). 10.3390/ijerph17082670 PMC7215880 32295024

[8] Liu Q, Pan H, Wu Y. Migration Status, Internet Use, and Social Participation among Middle-aged and older adults in China: consequences for Depression. Int J Environ Res Public Health. 2020;17(16). 10.3390/ijerph17166007 PMC7459605 32824867

[9] Miller LM, Steele JS, Wu CY, Kaye J, Dodge HH, Gonzales MM, Lyons KS. Depressive symptoms in older adult couples: associations with dyadic physical health, social engagement, and close friends. Front Psychiatry. 2022;13:989182. 36177214 10.3389/fpsyt.2022.989182 PMC9513127

[10] Wu HY, Chiou AF. Social media usage, social support, intergenerational relationships, and depressive symptoms among older adults. Geriatr Nurs. 2020;41(5):615–21. 32268948 10.1016/j.gerinurse.2020.03.016

[11] Yuen HK, Huang P, Burik JK, Smith TG. Impact of participating in volunteer activities for residents living in long-term-care facilities. Am J Occup Ther. 2008;62(1):71–6. 18254433 10.5014/ajot.62.1.71

[12] Ryu E, Jenkins GD, Wang Y, Olfson M, Talati A, Lepow L, Coombes BJ, Charney AW, Glicksberg BS, Mann JJ, et al. The importance of social activity to risk of major depression in older adults. Psychol Med. 2023;53(6):2634–42. 34763736 10.1017/S0033291721004566 PMC9095757

[13] Douglas H, Georgiou A, Westbrook J. Social participation as an indicator of successful aging: an overview of concepts and their associations with health. Aust Health Rev. 2017;41(4):455–62. 27712611 10.1071/AH16038

[14] Croezen S, Avendano M, Burdorf A, van Lenthe FJ. Social participation and depression in old age: a fixed-effects analysis in 10 European countries. Am J Epidemiol. 2015;182(2):168–76. 26025236 10.1093/aje/kwv015 PMC4493978

[15] Hofer M, Hargittai E. Online social engagement, depression, and anxiety among older adults. New Media Soc. 2024;26(1):113–30. 10.1177/14614448211054377

[16] Min J, Ailshire J, Crimmins EM. Social engagement and depressive symptoms: do baseline depression status and type of social activities make a difference? Age Ageing. 2016;45(6):838–43. 27496942 10.1093/ageing/afw125 PMC6312002

[17] Won S, Kim H. Social participation, health-related behavior, and depression of older adults living alone in Korea. Asian Soc Work Policy Rev. 2020;14(1):61–71. 10.1111/aswp.12193

[18] Hong SI, Hasche L, Bowland S. Structural relationships between social activities and longitudinal trajectories of depression among older adults. Gerontologist. 2009;49(1):1–11. 19362999 10.1093/geront/gnp006 PMC4047289

[19] van Hees SGM, van den Borne BHP, Menting J, Sattoe JNT. Patterns of social participation among older adults with disabilities and the relationship with well-being: a latent class analysis. Arch Gerontol Geriatr. 2020;86:103933. 31542633 10.1016/j.archger.2019.103933

[20] Choi J, Choi S, Song K, Baek J, Kim H, Choi M, Kim Y, Chu SH, Shin J. Everyday digital literacy questionnaire for older adults: Instrument Development and Validation Study. J Med Internet Res. 2023;25:e51616. 38095999 10.2196/51616 PMC10755654

[21] Kang YW, Jahng SM, Kim SY. Korean Dementia Association: Korean-Mini Mental State Examination, 2nd Edition (K-MMSE ~ 2) user’s guide. 2020. 10.12779/dnd.2020.19.4.161 PMC7781735 33377669

[22] Korea Employment Information Service: Korean Longitudinal Study of Ageing (KLoSA). Korea Employment Information Service: Chungcheongbuk-do Republic of Korea. 2020. https://survey.keis.or.kr/klosa/klosaque/List.jsp Accessed 12 Apr 2024.

[23] Radloff LS. The CES-D scale: a self-report depression scale for research in the general population. Appl Psychol Meas. 1977;1(3):385–401. 10.1177/014662167700100306

[24] Jeon G, Choi S, Yang B. Integrated Korean version of CES-D development. Kor J Psychol: Health. 2001;6(1):59–76.

[25] Vilagut G, Forero CG, Barbaglia G, Alonso J. Screening for Depression in the General Population with the Center for epidemiologic studies Depression (CES-D): a systematic review with Meta-analysis. PLoS ONE. 2016;11(5):e0155431. 27182821 10.1371/journal.pone.0155431 PMC4868329

[26] Chan E, Procter-Gray E, Churchill L, Cheng J, Siden R, Aguirre A, Li W. Associations among living alone, social support and social activity in older adults. AIMS Public Health. 2020;7(3):521–34. 32968675 10.3934/publichealth.2020042 PMC7505797

[27] Chen J, Zeng Y, Fang Y. Effects of social participation patterns and living arrangement on mental health of Chinese older adults: a latent class analysis. Front Public Health. 2022;10:915541. 35991050 10.3389/fpubh.2022.915541 PMC9390803

[28] Jang Y, Chiriboga DA. Social activity and depressive symptoms in Korean American older adults: the conditioning role of acculturation. J Aging Health. 2011;23(5):767–81. 21273501 10.1177/0898264310396214 PMC5788311

[29] Richard L, Gauvin L, Gosselin C, Laforest S. Staying connected: neighbourhood correlates of social participation among older adults living in an urban environment in Montreal, Quebec. Health Promot Int. 2009;24(1):46–57. 19098293 10.1093/heapro/dan039 PMC5167566

[30] Buchman AS, Boyle PA, Wilson RS, Fleischman DA, Leurgans S, Bennett DA. Association between late-life social activity and motor decline in older adults. Arch Intern Med. 2009;169(12):1139–46. 19546415 10.1001/archinternmed.2009.135 PMC2775502

[31] Ministry of Health and Welfare. 2020 National survey of Korean older adults. Ministry of Health and Welfare, Korea Institute for Health and Social Affairs: Sejong, Republic of Korea. 2020.

[32] Won CW, Yang KY, Rho YG, Kim S, Lee E-J, Yoon J, Cho K, Shin H, Cho BR, Oh J, et al. The development of Korean activities of daily living (K-ADL) and Korean instrumental activities of daily living (K-IADL) scale. Ann Geriatr Med Res. 2002;6(2):107–20.

[33] Won CW, Rho YG, Kim SY, Cho BR, Lee YS. The validity and reliability of Korean activities of Daily Living (K-ADL) scale. Ann Geriatr Med Res. 2002;6(2):98–106.

[34] Won CW, Rho YG, Sunwoo D, Lee YS. The validity and reliability of Korean Instrumental activities of Daily Living (K-IADL) scale. Ann Geriatr Med Res. 2002;6(4):273–80.

[35] Ware J Jr., Kosinski M, Keller SD. A 12-Item short-form Health Survey: construction of scales and preliminary tests of reliability and validity. Med Care. 1996;34(3):220–33. 8628042 10.1097/00005650-199603000-00003

[36] Zimet GD, Dahlem NW, Zimet SG, Farley GK. The multidimensional scale of perceived social support. J Pers Assess. 1988;52(1):30–41. 10.1207/s15327752jpa5201_2 2280326

[37] Shin JS, Lee YB. The effects of social supports on psychosocial well-being of the unemployed. Korean J Soc Welf. 1999;37:241–69.

[38] Weller BE, Bowen NK, Faubert SJ. Latent class analysis: a guide to best practice. J Black Psychol. 2020;46(4):287–311. 10.1177/0095798420930932

[39] Nylund-Gibson K, Choi AY. Ten frequently asked questions about latent class analysis. Transl Issues Psychol Sci. 2018;4(4):440–61. 10.1037/tps0000176

[40] Wang MC, Deng Q, Bi X, Ye H, Yang W. Performance of the entropy as an index of classification accuracy in latent profile analysis: a Monte Carlo simulation study. Acta Psychol Sin. 2017;49(11):1473–82. 10.3724/SP.J.1041.2017.01473

[41] Celeux G, Soromenho G. An entropy criterion for assessing the number of clusters in a mixture model. J Classif. 1996;13:195–212. 10.1007/BF01246098

[42] Williams GA, Kibowski F. Latent class analysis and latent profile analysis. In: *Handbook of methodological approaches to community-based research: Qualitative, quantitative, and mixed methods* 2016: 143–151.

[43] Asparouhov T, Muthén B. Auxiliary variables in mixture modeling: a 3-step approach using M plus. Struct Equ Model. 2014;21(3):329–41. 10.1080/10705511.2014.915181

[44] Cole MG, Dendukuri N. Risk factors for depression among elderly community subjects: a systematic review and meta-analysis. Am J Psychiatry. 2003;160(6):1147–56. 12777274 10.1176/appi.ajp.160.6.1147

[45] Djernes JK. Prevalence and predictors of depression in populations of elderly: a review. Acta Psychiatr Scand. 2006;113(5):372–87. 16603029 10.1111/j.1600-0447.2006.00770.x

[46] Vink D, Aartsen MJ, Schoevers RA. Risk factors for anxiety and depression in the elderly: a review. J Affect Disord. 2008;106(1–2):29–44. 17707515 10.1016/j.jad.2007.06.005

[47] Catalan-Matamoros D, Gomez-Conesa A, Stubbs B, Vancampfort D. Exercise improves depressive symptoms in older adults: an umbrella review of systematic reviews and meta-analyses. Psychiatry Res. 2016;244:202–9. 27494042 10.1016/j.psychres.2016.07.028

[48] Maier A, Riedel-Heller SG, Pabst A, Luppa M. Risk factors and protective factors of depression in older people 65+. A systematic review. PLoS ONE. 2021;16(5):e0251326. 33983995 10.1371/journal.pone.0251326 PMC8118343

[49] Jeon GS, Choi K. Purposes of Internet Use and its impacts on physical and psychological health of Korean older adults. Healthc (Basel) 2024, 12(2). 10.3390/healthcare12020244 PMC10815879 38255131

[50] Kim J, Lee HY, Christensen MC, Merighi JR. Technology Access and Use, and their associations with Social Engagement among older adults: do women and men Differ? J Gerontol B Psychol Sci Soc Sci. 2017;72(5):836–45. 28073816 10.1093/geronb/gbw123

[51] Lee H, Lim JA, Nam HK. Effect of a Digital Literacy Program on Older Adults’ Digital Social Behavior: A Quasi-Experimental Study. Int J Environ Res Public Health 2022, 19(19). 10.3390/ijerph191912404 PMC9564917 36231707

[52] Ngiam NHW, Yee WQ, Teo N, Yow KS, Soundararajan A, Lim JX, Lim HA, Tey A, Tang KWA, Tham CYX, et al. Building Digital Literacy in older adults of low socioeconomic status in Singapore (Project Wire Up): Nonrandomized Controlled Trial. J Med Internet Res. 2022;24(12):e40341. 36459398 10.2196/40341 PMC9758632

[53] Zapletal A, Wells T, Russell E, Skinner MW. On the triple exclusion of older adults during COVID-19: technology, digital literacy and social isolation. Soc Sci Humanit Open. 2023;8(1):100511. 37021073 10.1016/j.ssaho.2023.100511 PMC10060191

[54] Zhao W, Kelly RM, Rogerson MJ, Waycott J. Understanding older adults’ participation in Online Social activities: lessons from the COVID-19 pandemic. Proc ACM Hum Comput Interact. 2022;6(CSCW2):1–26.37360538

[55] Mubarak F, Suomi R. Elderly Forgotten? Digital Exclusion in the information age and the Rising Grey Digital divide. Inquiry. 2022;59:469580221096272. 35471138 10.1177/00469580221096272 PMC9052810

[56] Kania-Lundholm M, Torres S. The divide within: older active ICT users position themselves against different ‘Others’. J Aging Stud. 2015;35:26–36. 26568212 10.1016/j.jaging.2015.07.008

[57] Schreuers K, Quan-Haase A, Martin K. Problematizing the digital literacy paradox in the context of older adults’ ICT use: aging, media discourse, and self-determination. Can J Commun. 2017;42(2):1–34.

[58] Rolandi E, Vaccaro R, Abbondanza S, Casanova G, Pettinato L, Colombo M, Guaita A. Loneliness and Social Engagement in older adults based in Lombardy during the COVID-19 lockdown: the Long-Term effects of a course on Social networking sites Use. Int J Environ Res Public Health 2020, 17(21). 10.3390/ijerph17217912 PMC7662584 33126634

[59] Leung AY, Molassiotis A, Carino DA. A challenge to healthy aging: limited social participation in Old Age. Aging Dis. 2021;12(7):1536–8. 34631202 10.14336/AD.2021.02018 PMC8460313

[60] King AC. Interventions to promote physical activity by older adults. J Gerontol Biol Sci Med Sci. 2001;56(Spec 2):36–46. 10.1093/gerona/56.suppl_2.36 11730236

[61] Valdes-Badilla PA, Gutierrez-Garcia C, Perez-Gutierrez M, Vargas-Vitoria R, Lopez-Fuenzalida A. Effects of physical activity Governmental Programs on Health Status in Independent older adults: a systematic review. J Aging Phys Act. 2019;27(2):265–75. 29989461 10.1123/japa.2017-0396

[62] Fern AK. Benefits of physical activity in older adults: programming modifications to enhance the exercise experience. ACSM Health Fit J. 2009;13(5):12–6. 10.1249/FIT.0b013e3181b46b23

[63] Gill K, Overdorf V. Incentives for exercise in younger and older women. J Sport Behav. 1994;17(2):87–98.

[64] King AC, Rejeski WJ, Buchner DM. Physical activity interventions targeting older adults. A critical review and recommendations. Am J Prev Med. 1998;15(4):316–33. 9838975 10.1016/S0749-3797(98)00085-3

[65] Zuo Y, Ma Y, Zhang M, Wu X, Ren Z. The impact of sharing physical activity experience on social network sites on residents’ social connectedness:a cross-sectional survey during COVID-19 social quarantine. Global Health. 2021;17(1):10. 33430894 10.1186/s12992-021-00661-z PMC7797884

